# Detection of SARS-CoV-2 at the point of care

**DOI:** 10.4155/bio-2021-0078

**Published:** 2021-07-22

**Authors:** Michael J Loeffelholz, Yi-Wei Tang

**Affiliations:** ^1^Cepheid, Sunnyvale, CA, USA; ^2^Danaher Diagnostic Platform, Shanghai, China

**Keywords:** COVID-19, home testing, nucleic acid amplification testing, POCT, pooled specimen screening, rapid antigen testing, SARS-CoV-2

## Abstract

Severe acute respiratory syndrome coronavirus 2 (SARS-CoV-2) is the etiologic agent of COVID-19. Testing for SARS-CoV-2 infection is a critical element of the public health response to COVID-19. Point-of-care (POC) tests can drive patient management decisions for infectious diseases, including COVID-19. POC tests are available for the diagnosis of SARS-CoV-2 infections and include those that detect SARS-CoV-2 antigens as well as amplified RNA sequences. We provide a review of SARS-CoV-2 POC tests including their performance, settings for which they might be used, their impact and future directions. Further optimization and validation, new technologies as well as studies to determine clinical and epidemiological impact of SARS-CoV-2 POC tests are needed.

## Current global COVID-19 situation, & role of laboratory testing

Severe acute respiratory syndrome coronavirus (SARS-CoV-2), the etiologic agent of COVID-19, was first detected in Wuhan, Hubei Province, China in December of 2019 [1]. The rapid increase and spread in reported cases triggered the World Health Organization to declare COVID-19 to be a global pandemic on 11 March 2020. As of 20 April 2021, over 140 million confirmed COVID-19 cases and over 3 million deaths have been reported globally (https://www.who.int/emergencies/diseases/novel-coronavirus-2019).

Testing for SARS-CoV-2 infection is a critical component of the public health response to COVID-19, in part because asymptomatic infections are common and contribute to COVID-19 transmission [2,3]. Testing identifies infected individuals to be quarantined or isolated for infection control purposes [4], (1) impacts patient management decisions [5] (2), and guides therapeutic strategies (https://www.covid19treatmentguidelines.nih.gov/). Antivirals and monoclonal antibody therapies are available for COVID-19, and their initiation is dependent upon positive results of SARS-CoV-2 testing.

## Laboratory tests for SARS-CoV-2

Laboratory tests for SARS-CoV-2 infection include nucleic acid amplification, antigen detection and antibody or serologic tests [6–8]. Nucleic acid amplification tests (NAATs) and antigen tests provide diagnosis of acute infection when viral titers are highest. In addition, NAATs can reliably detect asymptomatic and pre-symptomatic infections [9,10], including in pooled specimens [10–14] due to their high sensitivity [15]. Because of their high sensitivity and specificity, NAATs are considered the gold standard for diagnostic detection of SARS-CoV-2 (https://www.cdc.gov/coronavirus/2019-ncov/lab/resources/antigen-tests-guidelines.html). Because it may take 10 or more days after symptom onset for development of detectable levels of antibodies to SARS-CoV-2 to occur [16,17], serologic tests should not be used alone to diagnose acute COVID-19 [18,19].

## Point-of-care laboratory tests for SARS-CoV-2 & their regulation in USA

The current widely accepted definition of point of care (POC) testing includes testing that occurs at or near the point of patient care, such that the results drive patient care decisions made during that encounter [20,21]. POC tests are performed in a variety of settings including physician offices, emergency department, urgent care facilities, school health clinics and pharmacies. Some POC tests are approved, cleared or authorized by regulatory bodies to be performed by non-laboratory professionals [22,23]. Recently, the COVID-19 pandemic has shined a spotlight on Clinical and Laboratory Improvement Amendments (CLIA)-waived diagnostic testing. Some SARS-CoV-2 tests have received Emergency Use Authorization (EUA) from the US FDA for use in CLIA-waived testing sites. Waived testing is defined by CLIA as simple tests that carry a low risk for an incorrect result. Waived tests can be performed without the need for the conduct of more stringent quality standards imposed by CLIA for non-waived testing. Both waived and non-waived tests can be performed at the POC. But the complexity of tests performed at a testing site is determined by its type of CLIA certificate; a testing site with a certificate of waiver can perform only waived tests. Under CLIA, testing sites with a certificate of waiver are generally exempt from most US regulatory oversight requirements for non-waived laboratories, including personnel qualifications, training, test method verification and proficiency testing or external quality assessment. However, some accreditation agencies in the US and local or state governments may have more strict quality requirements for POC sites that perform testing for SARS-CoV-2. For a list of SARS-CoV-2 antigen tests with EUA for settings operating under a CLIA Certificate of Waiver, see Table 1. Additional FDA authorizations for at-home use are described separately.

**Table 1.** Rapid antigen tests with US FDA EUA for use at settings operating under a CLIA certificate of waiver.

Assay name	Manufacturer	Assay format	SARS-CoV-2 limit of detection^†^	Published studies describing performance	Published outcome studies	Ref.
BD Veritor SARS-CoV-2; BD Veritor SARS-CoV-2 & Flu A + B	Becton, Dickinson and Company	Digital chromatographic immunoassay; instrument read	2.8 × 10^2^ TCID50/ml		None	[24,25]
BinaxNOW COVID-19	Abbott Diagnostics Scarborough, Inc.	Lateral flow; visual read	140.6 TCID50/ml		None	[26–32]
CareStart COVID-19	Access Bio, Inc.	Lateral flow; visual read	8 × 10^2^ TCID50/ml	None	None	
Clip COVID	Luminostics, Inc.	Lateral flow immunoluminescent; instrument read	0.88 × 10^2^ TCID50/ml	None	None	
Ellume COVID-19	Ellume Limited	Lateral flow; fluorescence; instrument read	10^3.80^ TCID50/ml	None	None	
LumiraDx SARS-CoV-2	LumiraDx UK Ltd.	Microfluidic immunofluorescence; instrument read	32 TCID50/ml		None	[33,34]
QuickVue COVID-19 and QuickVue SARS	Quidel Corporation	Lateral flow; visual read	1.91 × 10^4^ TCID50/ml	None	None	
Sofia SARS Antigen FIA	Quidel Corporation	Lateral flow; fluorescence; instrument read	1.13 × 10^2^ TCID50/ml		None	[35–38]
Sofia 2 Flu + SARS	Quidel Corporation	Lateral flow; fluorescence; instrument read	91.7 TCID50/ml		None	[24]
Status COVID-19/Flu	Princeton BioMeditech Corp.	Lateral flow; visual read	2.7 × 10^3^ TCID50/ml	None	None	

† Information from manufacturers' instructions for use available at [39].

Commercially available SARS-CoV-2 NAATs and antigen tests are available in both high throughput batch-based format often employing large instrumentation, and single-use portable sample-to-answer format that can be performed on demand for fast results. Rapid, single-use tests are often used at the POC. The World Health Organization (WHO) developed the ASSURED criteria as a standard for rapid tests for detection of sexually transmitted infections in resource-limited settings [40]. These criteria can be applied to POC tests for all infectious diseases, including COVID-19. The ASSURED criteria describe the most desirable attributes of POC tests, and are **A**ffordable, **S**ensitive, **S**pecific, **U**ser-friendly, **R**apid and robust, **E**quipment-free and **D**eliverable to end-users [41]. Some of these criteria, such as equipment-free, are more relevant for resource-limited, remote settings with unreliable electrical power. But affordability, test accuracy and rapid time to result are attributes of POC that are important universally.

## POC rapid antigen tests

Rapid antigen tests with US FDA EUA as of 4 April 2021, for use in patient care settings operating under a CLIA certificate of waiver are listed in Table 1. In general, the analytical sensitivity, or lower limit of detection of rapid antigen tests is several logs less than that of the most sensitive NAATs. This consistently translates in lower diagnostic sensitivity, or positive agreement with NAATs [42]. In direct comparisons against RT-PCR, rapid antigen tests were most likely to produce false-negative results from specimens with late RT-PCR cycle threshold values (>30) corresponding with lower viral loads.

An evaluation of the BD Veritor SARS-CoV-2 test (Becton, Dickinson and Company, MD, USA) in a symptomatic population with median age of 43 years showed positive percent agreement (PPA) with RT-PCR ranged from 87.5% for specimens collected within 1 day after symptom onset, to 76.3% for specimens collected 7 days after symptom onset [24]. Most specimens with false-negative Veritor results had RT-PCR cycle threshold (Ct) values of 25 or greater. The same study also directly compared the Veritor assay with the Sofia 2 Flu + SARS assay (Quidel Corporation, CA, USA), and showed high (>97%) PPA and negative percent agreement (NPA). Another evaluation of the Veritor SARS-CoV-2 test, also performed in a symptomatic population with a similar median age, showed Veritor sensitivity of 66.4% compared with an EUA RT-PCR test (Simplexa COVID-19; Diasorin Molecular LLC, CA, USA) [25]. Again, specimens with discordant negative Veritor results had higher Ct values by RT-PCR.

Several studies have evaluated the performance of the BinaxNOW test (Abbott Diagnostics Scarborough, Inc., ME, USA) [26–32]. Prince-Guerra reported high specificity of BinaxNOW in a population with median age of 41 years, but sensitivities of 35.8 and 64.2% from asymptomatic and symptomatic patients, respectively [26]. When viral culture was positive, BinaxNOW sensitivity increased to 78.6 and 92.6% among asymptomatic and symptomatic subjects, respectively. James *et al.* reported slighter higher sensitivity of BinaxNOW than reported by Prince-Guerra, (51.6 and 83.3% in asymptomatic and symptomatic persons, respectively) [30] but their study was conducted in an entirely adult population. Another study reported BinaxNOW sensitivity of specificities of 93.3 and 99.9%, respectively, in subjects with high viral loads (RT-PCR Ct values <30) [27]. Pollock *et al.* evaluated the performance of BinaxNOW in a community drive-through testing site. BinaxNOW specificity was high in both adults and children. Sensitivities were 96.5 and 84.6%, compared with RT-PCR, in adults and children, respectively, within 7 days of symptom onset. In asymptomatic adults and children, sensitivities were 70.2 and 65.4%, respectively [31]. As reported elsewhere, sensitivity was highest (95.8%) when RT-PCR Ct values were ≤30. Okoye *et al.* evaluated BinaxNOW in an asymptomatic university setting (mean age 24 years) and found 53.3% sensitivity compared with RT-PCR.

As described above, the Sofia 2 assay was shown to have high agreement with BD Veritor assay for SARS-CoV-2 antigen detection. Another study reported Sofia PPA with Aptima SARS-CoV-2 TMA test (Hologic, Inc., CA, USA) of 82% in patients presenting within 5 days of symptom onset, and 54.5% when specimens were collected greater than 5 days after symptom onset [32]. Unlike other studies which showed lower sensitivity/positive agreement for antigen tests performed in children, the positive agreement of Sofia did not vary substantially between children and adults. Evaluation of Sofia at a university setting (primarily students) showed sensitivities of 80 and 41.2%, compared with RT-PCR, in symptomatic and asymptomatic persons, respectively [35]. Kohmer *et al.* compared the LumiraDx SARS-CoV-2 test (LumiraDx UK Ltd, Alloa, UK) to quantitative RT-PCR [33]. Among residents of a shared living facility, screened without regard for clinical symptoms, LumiraDx sensitivity and specificity were 50 and 100%, respectively. Of RT-PCR positive specimens containing ≥6 log10 RNA copies/ml, LumiraDx sensitivity was 100%. Drain *et al.* compared LumiraDx to the cobas 6800 RT-PCR test (Roche Molecular Systems, NJ, USA) in a multi-site study [34]. Most subjects (81%) were symptomatic and the mean age was 34 years. Overall, the LumiraDx assay had a reported sensitivity of 97.6% and specificity of 96.6% up to 12 days post symptom onset for nasal swab samples.

A recent meta-analysis reported the sensitivities of rapid POC antigen tests [43]. Time post symptom onset and viral loads [as estimated by PCR cycle threshold (Ct) values] impacted the sensitivity of antigen tests. Average sensitivity of antigen tests was higher when specimens were collected during the first week compared with second week post symptom onset (78.3 vs 51.0%, respectively). Average sensitivity in specimens with PCR Ct values ≤25 was 94.5%, compared with 40.7% in specimens with Ct values >25. Unfortunately, we were unable to find any published peer-review studies evaluating the outcomes provided by rapid antigen tests performed at the POC.

## POC NAATs

NAATs, particularly RT-PCR, are considered the gold-standard for laboratory diagnosis of SARS-CoV-2 infection because of their high sensitivity and specificity. However, some rapid NAATs are less sensitive than RT-PCR-based POC tests. Table 2 provides a list of SARS-CoV-2 NAATs with EUA for use in CLIA-waived testing sites, as of 3 April 2021. Additional authorizations for at-home use are described separately.

**Table 2.** Nucleic acid amplification tests with US FDA EUA for use at settings operating under a CLIA certificate of waiver.

Assay name	Manufacturer	Assay format	SARS-CoV-2 limit of detection^†^	Published studies describing performance	Published outcome studies	Ref.
Accula™ SARS-CoV-2	Mesa Biotech, Inc.	RT-PCR; lateral flow; visual read	150 copies/ml		None	[44]
BioFire^®^ Respiratory Panel 2.1-EZ	Biofire Diagnostics, LLC	Reverse transcription; nested multiplex PCR; instrument read	500 copies/ml	No evaluations of 2.1-EZ. Studies of *de novo* 510(k)-cleared Respiratory Panel 2.1 for non-waived testing laboratories	None	[45,46]
cobas^®^ SARS-CoV-2 & Influenza A/B; cobas Liat System	Roche Molecular Systems, Inc.	RT-PCR; instrument read	0.012 TCID50/ml		None	[47]
Cue™ COVID-19	Cue Health, Inc.	Reverse transcription; isothermal amplification; mobile smart device read	20 genome copies/sample wand		None	[48]
ID NOW COVID-19	Abbott Diagnostics Scarborough, Inc.	Reverse transcription; isothermal amplification; instrument read	125 genome equivalents/ml			[30,43,49–63]
Lucira™ COVID-19	Lucira Health, Inc.	Reverse transcription; isothermal amplification; disposable device read	2700 copies per swab	None	None	
Xpert^®^ Xpress SARS-CoV-2	Cepheid	RT-PCR; instrument read	0.0200 PFU/ml			[43,53,56,59,60,62,64–82,83,84]
Xpert Xpress SARS-CoV-2/Flu/RSV	Cepheid	RT-PCR; instrument read	131 copies/ml		None	[85,86]
Visby Medical™ COVID-19	Visby Medical, Inc.	RT-PCR; visual read	435 copies/swab	None	None	

† Information from manufacturers' instructions for use, available at [87].

Hogan *et al.* compared the Accula™ SARS-CoV-2 test (Mesa Biotech, Inc., CA, USA) to a laboratory developed RT-PCR test [44]. The Accula test had poor PPA of 68%, missing specimens containing low viral loads. We were unable to find published studies of the performance of the BioFire^®^ Respiratory Panel 2.1-EZ panel (BioFire Diagnostics, LLC, UT, USA). However, the non-waived version of the panel has been evaluated and demonstrated good agreement with laboratory-developed and EUA RT-PCR tests [45,46]. Analytically, the BioFire SARS-CoV-2 assay was less sensitive than the Xpert^®^ Xpress SARS-CoV-2 (Cepheid, CA, USA) and cobas tests, but more sensitive than ID NOW [46]. A multi-site study comparing the cobas Liat SARS-CoV-2 & influenza A/B nucleic acid test to the cobas 68/8800 SARS-CoV-2 test showed PPA and NPA for SARS-CoV-2 of 100 and 97.4%, respectively [47]. Donato *et al.* compared the Cue™ COVID-19 test (Cue Health Inc., CA, USA) to the Aptima SARS-CoV-2 on a Hologic Panther instrument. In a community drive through collection setting including both symptomatic and asymptomatic outpatients the overall PPA and NPA were 91.7 and 98.4%, respectively [48].

The ID NOW COVID-19 test (Abbott Diagnostics Scarborough, Inc.) has been extensively evaluated [30,43,49–62]. Analytically, ID NOW is substantially less sensitive than RT-PCR-based methods [62]. This lower analytical sensitivity translates into lower PPA compared with RT-PCR, which ranged from 48 to 94% in the cited studies. As expected, specimens positive by RT-PCR that were negative by ID NOW had higher Ct values (lower viral loads). In one study, ID NOW PPA was 34.3% among specimens with RT-PCR Ct values >30 [60]. Processing nasal swabs directly by the ID NOW assay, versus pre-elution in 3 ml of viral transport medium (VTM), has been recommended to improve sensitivity. However, two studies showed that direct processing of swabs actually decreased ID NOW PPA compared with swabs in VTM [53,88]. Studies showed high NPA of ID NOW, near or at 100%.

The Xpert Xpress SARS-CoV-2 test (Cepheid) has also been extensively evaluated [43,53,56,59,60,62,64–82]. Most studies have reported high sensitivity or PPA- >98%- compared with other RT-PCR-based tests. Lowe *et al.* reported 100% PPA between Xpress SARS-CoV-2 test and cobas among specimens with cobas Ct values between 30 and 33.9, but Xpress test failed to detect three of nine specimens with cobas Ct values ≥34 [67]. Most studies also report high NPA with other RT-PCR-based tests. When positive Xpress SARS-CoV-2 results were discordant with the comparator method, testing with a third highly sensitive method usually agreed with Xpress test [65,79]. Falasca *et al.* described specimens with late Xpress SARS-CoV-2 Ct values for the N2 target [73]. Authors attempted to concentrate specimens by centrifugation to increase the positive signal. This failed in some instances, but the relative centrifugal force used may have been insufficient to pellet free virus. Khoshchehreh *et al.* reported that Xpress SARS-CoV-2 results with late Ct values occurred in symptomatic patients with new COVID-19 diagnosis, and in asymptomatic patients with clinical findings consistent with sub-clinical disease [74]. In one study, the Xpert Xpress test was compared in parallel to two SARS-CoV-2 assays (BioGerm and Sansure) cleared by the Chinese National Medical Products Administration in China. The sensitivity of Xpress SARS-CoV-2 test was 100% compared with 96.15% for the BioGerm and 90% for the Sansure kits. The specificity was 100% for all three assays. The limit of detection is 100 copies/ml for Xpert Xpress and 500 copies/ml for the BioGerm kit and Sansure kit [66].

Recognizing that SARS-CoV-2 transmission may occur during seasonal influenza and concurrent with transmission of other respiratory viruses such as respiratory syncytial virus (RSV), several manufacturers have created multiplexed assays for simultaneous detection of SARS-CoV-2 and additional respiratory viruses. These include the BD Veritor SARS-CoV-2 & Flu A + B (Becton, Dickinson and Company), Sofia 2 Flu + SARS (Quidel Corporation), Status COVID-19/Flu (Princeton BioMeditech Corp.), BioFire^®^ Respiratory Panel 2.1-EZ (Biofire Diagnostics, LLC), cobas^®^ SARS-CoV-2 & Influenza A/B (Roche Molecular Systems, Inc.), and Xpert^®^ Xpress SARS-CoV-2/Flu/RSV (Cepheid) (Tables 1 & 2). The performance of the Xpert Xpress SARS-CoV-2/Flu/RSV test has been described in two studies [85,86]. PPA and NPA were high when compared with other RT-PCR based tests.

## Impact of SARS-CoV-2 NAATs performed at the POC

We identified three peer-reviewed studies that evaluated the impact of rapid NAAT performed at the POC, including one which involved the Xpert Xpress SARS-CoV-2 test. Hinson *et al.* performed the Xpress SARS-CoV-2 test on demand in the Emergency Department and compared impact with batch RT-PCR testing in a central laboratory [89]. Rapid testing at the POC was associated with a significant reduction in the time to removal from isolation for patients with negative test results, an increase in COVID-19 treatment capacity, and conservation of personal protective equipment. Another study compared the impact of a non-EUA NAAT performed at POC, to a batch-based RT-PCR performed in a central laboratory [89]. The time to result for the POC NAAT was significantly shorter than the standard lab RT-PCR, and POC testing resulted in faster time to final bed placement from the Emergency Room, increased isolation room availability, and faster patient discharge. Brendish *et al.* reported that time to arrival in a definitive clinical area was significantly shortened by rapid POC molecular testing [90].

Hengel *et al.* described a model for the implementation of decentralized COVID-19 POC testing in remote locations by use of the Cepheid GeneXpert^®^ platform, which has been successfully scaled up in remote Aboriginal and Torres Strait Islander communities across Australia. The analysis indicated that implementation of the decentralized POC testing model should be considered for communities in need, especially those that are undertested and socially vulnerable [91].

## At-home testing for SARS-CoV-2

SARS-CoV-2 tests with FDA authorization for use at home are primarily based on rapid isothermal amplification of nucleic acids or antigen detection. The advantages and limitations of these methods have been described [92]. At-home testing (specimen collection, test and result interpretation) is performed by an individual on their own without supervision of a trained health professional. For the purpose of this review, we considered at-home tests separately from POC tests, because they are not performed at the same location where care is provided. At-home test results do not guarantee initiation of treatment or other interventions. Nonetheless, at-home tests have the potential to broaden access to testing and increase testing rates [93,94]. To date the US FDA has granted EUA to five SARS-CoV-2 tests for at-home use. Four of these at-home tests have received EUA for over-the-counter (OCT) use without a prescription, and one has received EUA for prescription home testing. Rapid antigen tests with at-home OTC use authorization include the QuickVue^®^ At-Home OTC COVID-19 test (Quidel), BinaxNOW™ COVID-19 Antigen test (Abbott), and Ellume COVID-19 Home Test (Ellume Limited, East Brisbane, Australia). A single NAAT has received EUA for OTC at-home use: Cue™ COVID-19 Test (Cue Health). Finally, one NAAT has EUA for prescription home testing: Lucira™ COVID-19 All-In-One Test (Lucira Health, Inc., CA, USA).

## Challenges

While there are now many commercial options for SARS-CoV-2 POC testing, challenges remain. These challenges include maintaining a reliable supply chain, and a lack of healthcare infrastructure to manage a pandemic the scope of COVID-19, particularly in low and middle-income countries [95]. The accuracy of SARS-CoV-2 POC tests varies widely as described in this review and many published studies. Providing access to highly accurate primary testing is ideal, and assuring prompt confirmatory testing is critical when less accurate POC tests are used. It is important that providers understand the characteristics of the SARS-CoV-2 tests that they perform.

Rapid antigen SARS-CoV-2 tests are substantially less sensitive than most NAATs. POC rapid antigen tests have higher sensitivity when viral loads are high, allowing these tests to identify infected persons at highest risk of transmission. However, it is important to identify all infected persons, even those with low-to-moderate viral loads, as these infections represent opportunity for the virus to mutate. Additionally, identification and tracking all infections is necessary to have a full understanding of the COVID-19 pandemic epidemiology, and effectiveness of interventions. Cases with low to moderate viral loads could represent early infection, or pre-syndromic cases. Identifying and isolating these persons before they have high viral loads and are most contagious is important to control the spread of SARS-CoV-2.

Additionally, the global COVID-19 public health emergency will eventually end and EUAs expire, but the need for SARS-CoV-2 testing will remain. It is crucial that manufacturers of SARS-CoV-2 tests have a long-term strategy for product registration beyond EUA. Finally, studies evaluating the impact of NAATs versus rapid antigen tests performed at the POC are urgently needed. The higher sensitivity of SARS-CoV-2 NAATs may provide greater impact than rapid antigen tests, as has been shown for influenza virus testing performed at the POC [96]. While more impact studies including cost-benefit analysis are needed for SARS-CoV-2 POC tests, the decentralized POC testing model should be part of the core global response toward suppressing COVID-19.

## Conclusion

There are a number of new point-of-care tests for SARS-CoV-2. The performance of these tests varies greatly. Rapid antigen SARS-CoV-2 tests and some rapid NAATs are substantially less sensitive than most RT-PCR based NAATs. While all POC SARS-CoV-2 tests are able to identify most highly infectious cases when viral loads are high, sensitive tests that allow detection of early pre-symptomatic infection prior to peak contagiousness are also important to prevent disease transmission. Detection of asymptomatic infections, often with low viral loads, is crucial for bringing the COVID-19 pandemic to an end as these infections represent opportunity for the virus to mutate. Studies evaluating the impact of NAATs versus rapid antigen tests performed at the POC are urgently needed. While more impact studies including cost-benefit analysis are needed for SARS-CoV-2 POC tests, the decentralized POC testing model should be part of the core global response towards combatting COVID-19.

## Future perspective

The scope of the COVID-19 pandemic and the impact on testing access has driven heightened interest in new and portable diagnostic technologies. A challenge is the often-conflicting test characteristics of speed (time to result) and sensitivity; as we attempt to make tests faster we often must compromise on sensitivity. Portability, an essential characteristic of POC tests, is also a challenge. Nanotechnology, including novel sensors to detect low concentrations of SARS-CoV-2 proteins, supported by artificial intelligence and integration within the internet for wireless operation and connectivity has the potential to revolutionize POC diagnostics [97–99]. Another technology which could contribute to near patient testing is digital PCR (dPCR). While its attributes are mainly related to target quantification, dPCR increases the signal-to-noise ratio, making it highly sensitive and amenable to a rapid time to result [100,101].

**Executive summary**

- Laboratory tests for SARS-CoV-2 include those performed at or near the point-of-care (POC). In USA, most SARS-CoV-2 tests have received emergency use authorization (EUA) from US FDA. SARS-CoV-2 POC tests designed to detect acute infection include those that detect SARS-CoV-2 antigen or nucleic acid.
- At the time of writing, only three studies evaluated the impact of POC NAATs, and these studies demonstrated positive impact including more rapid isolation of hospitalized patients and appropriate use of PPE. There have been no published studies evaluating the impact of POC antigen tests.
- At-home SARS-CoV-2 tests are now available. These have the potential of increasing access to testing.
- POC SARS-CoV-2 tests are now widely used, and while their rapid time to result has positive impact, challenges remain. These challenges include testing access in many parts of the world, adequate public health infrastructure and accurate performance of many POC tests. New technologies have the potential of increasing the sensitivity of POC tests that detect SARS-CoV-2 antigens.

## References

[1] Lu H, Stratton CW, Tang YW. Outbreak of pneumonia of unknown etiology in Wuhan, China: the mystery and the miracle. J. Med. Virol. 92(4), 401–402 (2020). 31950516 10.1002/jmv.25678 PMC7166628

[2] Han D, Li R, Han Y, Zhang R, Li J. COVID-19: insight into the asymptomatic SARS-COV-2 infection and transmission. Int J Biol Sci. 16(15), 2803–2811 (2020). 33061797 10.7150/ijbs.48991 PMC7545704

[3] Vandenberg O, Martiny D, Rochas O, van Belkum A, Kozlakidis Z. Considerations for diagnostic COVID-19 tests. Nat Rev Microbiol. 19(3), 171–183 (2021). 33057203 10.1038/s41579-020-00461-z PMC7556561

[4] Wake RM, Morgan M, Choi J, Winn S. Reducing nosocomial transmission of COVID-19: implementation of a COVID-19 triage system. Clin Med (Lond). 20(5), e141–e145 (2020). 32788160 10.7861/clinmed.2020-0411 PMC7539706

[5] Nielsen Jeschke K, Bonnesen B, Hansen EF Guideline for the management of COVID-19 patients during hospital admission in a non-intensive care setting. Eur Clin Respir J. 7(1), 1761677 (2020). . 33224450 10.1080/20018525.2020.1761677 PMC7655082

[6] Loeffelholz MJ, Tang YW. Laboratory diagnosis of emerging human coronavirus infections - the state of the art. Emerg Microbes Infect. 9(1), 747–756 (2020). 32196430 10.1080/22221751.2020.1745095 PMC7172701

[7] Tang YW, Schmitz JE, Persing DH, Stratton CW. Laboratory Diagnosis of COVID-19: Current Issues and Challenges. J. Clin. Microbiol. 58(6), e00512–00520 (2020). 32245835 10.1128/JCM.00512-20 PMC7269383

[8] Chauhan N, Soni S, Gupta A, Jain U. New and developing diagnostic platforms for COVID-19: a systematic review. Expert Rev Mol Diagn. 20(9), 971–983 (2020). 32896179 10.1080/14737159.2020.1816466

[9] Byrne AW, McEvoy D, Collins AB Inferred duration of infectious period of SARS-CoV-2: rapid scoping review and analysis of available evidence for asymptomatic and symptomatic COVID-19 cases. BMJ Open 10(8), e039856 (2020). 10.1136/bmjopen-2020-039856 PMC7409948 32759252

[10] Pan Y, Zhang D, Yang P, Poon LLM, Wang Q. Viral load of SARS-CoV-2 in clinical samples. Lancet Infect Dis. 20(4), 411–412 (2020). 32105638 10.1016/S1473-3099(20)30113-4 PMC7128099

[11] Eberhardt JN, Breuckmann NP, Eberhardt CS. Multi-stage group testing improves efficiency of large-scale COVID-19 screening. J Clin Virol. 128, 104382 (2020). 32388468 10.1016/j.jcv.2020.104382 PMC7177109

[12] Barat B, Das S, De Giorgi V Pooled Saliva Specimens for SARS-CoV-2 Testing. J. Clin. Microbiol. 59(3), e02486–02420 (2021). 33262219 10.1128/JCM.02486-20 PMC8106731

[13] More S, Narayanan S, Patil G Pooling of nasopharyngeal swab samples to overcome a global shortage of real-time reverse transcription-PCR COVID-19 test kits. J. Clin. Microbiol. 59(4), e01295–01220 (2021). 33500363 10.1128/JCM.01295-20 PMC8092752

[14] Cherif A, Grobe N, Wang X, Kotanko P. Simulation of pool testing to identify patients with coronavirus disease 2019 under conditions of limited test availability. JAMA Netw Open 3(6), e2013075 (2020). 32573706 10.1001/jamanetworkopen.2020.13075 PMC7315509

[15] Li H, Sun K, Persing DH, Tang YW, Shen D. Real-time screening of specimen pools for coronavirus disease 2019 (COVID-19) infection at Sanya Airport, Hainan Island, China. Clin. Infect. Dis. 30(10), (2020). 10.1093/cid/ciaa1074 PMC7543279 32997114

[16] Long QX, Liu BZ, Deng HJ Antibody responses to SARS-CoV-2 in patients with COVID-19. Nat. Med. 26(6), 845–848 (2020). 32350462 10.1038/s41591-020-0897-1

[17] Wölfel R, Corman VM, Guggemos W Virological assessment of hospitalized patients with COVID-2019. Nature 581(7809), 465–469 (2020). 32235945 10.1038/s41586-020-2196-x

[18] Theel ES, Slev P, Wheeler S, Couturier MR, Wong SJ, Kadkhoda K. The role of antibody testing for SARS-CoV-2: is there one? J. Clin. Microbiol. 58(8), e00797–00720 (2020). 32350047 10.1128/JCM.00797-20 PMC7383527

[19] Wang H, Ai J, Loeffelholz MJ, Tang YW, Zhang W. Meta-analysis of diagnostic performance of serology tests for COVID-19: impact of assay design and post-symptom-onset intervals. Emerg Microbes Infect. 9(1), 2200–2211 (2020). 32962560 10.1080/22221751.2020.1826362 PMC7580610

[20] Chen H, Liu K, Li Z, Wang P. Point of care testing for infectious diseases. Clin. Chim. Acta 493, 138–147 (2019). 30853460 10.1016/j.cca.2019.03.008 PMC6462423

[21] Drancourt M, Michel-Lepage A, Boyer S, Raoult D. The Point-of-Care Laboratory in Clinical Microbiology. Clin. Microbiol. Rev. 29(3), 429–447 (2016). 27029593 10.1128/CMR.00090-15 PMC4861988

[22] Song Q, Sun X, Dai Z Point-of-care testing detection methods for COVID-19. Lab Chip. 21(9), 1634–1660 (2021). 33705507 10.1039/d0lc01156h

[23] El Jaddaoui I, Allali M, Raoui S A review on current diagnostic techniques for COVID-19. Expert Rev Mol Diagn. 21(2), 141–160 (2021). 33593219 10.1080/14737159.2021.1886927

[24] Young S, Taylor SN, Cammarata CL Clinical evaluation of BD Veritor SARS-CoV-2 point-of-care test performance compared to PCR-based testing and versus the Sofia 2 SARS antigen point-of-care test. J. Clin. Microbiol. 59(1), e02338–02320 (2020). 33023911 10.1128/JCM.02338-20 PMC7771450

[25] Kilic A, Hiestand B, Palavecino E. Evaluation of performance of the BD Veritor SARS-CoV-2 chromatographic immunoassay test in COVID-19 symptomatic patients. J. Clin. Microbiol. 26, 00260–00221 (2021). 10.1128/JCM.00260-21 PMC8091854 33637583

[26] Prince-Guerra JL, Almendares O, Nolen LD Evaluation of Abbott BinaxNOW rapid antigen test for SARS-CoV-2 infection at two community-based testing sites - Pima County, Arizona, November 3–17, 2020. MMWR Morb. Mortal. Wkly Rep. 70(3), 100–105 (2021). 33476316 10.15585/mmwr.mm7003e3 PMC7821766

[27] Pilarowski G, Lebel P, Sunshine S Performance characteristics of a rapid severe acute respiratory syndrome coronavirus 2 antigen detection assay at a public plaza testing site in San Francisco. J. Infect. Dis. 223(7), 1139–1144 (2021). 33394052 10.1093/infdis/jiaa802 PMC7799021

[28] Pilarowski G, Marquez C, Rubio L Field performance and public health response using the BinaxNOW TM Rapid SARS-CoV-2 antigen detection assay during community-based testing. Clin. Infect. Dis. 26(10), (2020). 10.1093/cid/ciaa1890 PMC7799223 33367619

[29] Perchetti GA, Huang ML, Mills MG, Jerome KR, Greninger AL. Analytical sensitivity of the Abbott BinaxNOW COVID-19 Ag Card. J. Clin. Microbiol. 59(3), e02880–02820 (2021). 33310764 10.1128/JCM.02880-20 PMC8106729

[30] James AE, Gulley T, Kothari A, Holder K, Garner K, Patil N. Performance of the BinaxNOW coronavirus disease 2019 (COVID-19) Antigen Card test relative to the severe acute respiratory coronavirus virus 2 (SARS-CoV-2) real-time reverse transcriptase polymerase chain reaction (rRT-PCR) assay among symptomatic and asymptomatic healthcare employees. Infect. Control Hosp. Epidemiol. 25, 1–3 (2021). 10.1017/ice.2021.20 PMC7870908 33487197

[31] Pollock NR, Jacobs JR, Tran K Performance and Implementation Evaluation of the Abbott BinaxNOW Rapid Antigen Test in a High-throughput Drive-through Community Testing Site in Massachusetts. J. Clin. Microbiol. 23, 00083–00021 (2021). 10.1128/JCM.00083-21 PMC8091851 33622768

[32] Okoye NC, Barker AP, Curtis K Performance characteristics of BinaxNOW COVID-19 antigen card for screening asymptomatic individuals in a university setting. J. Clin. Microbiol. 59(4), e03282–03220 (2021). 33509809 10.1128/JCM.03282-20 PMC8092740

[33] Kohmer N, Toptan T, Pallas C The comparative clinical performance of four SARS-CoV-2 rapid antigen tests and their correlation to infectivity *In Vitro*. J Clin Med. 10(2), 328 (2021). 33477365 10.3390/jcm10020328 PMC7830733

[34] Drain PK, Ampajwala M, Chappel C A rapid, high-sensitivity SARS-CoV-2 nucleocapsid immunoassay to aid diagnosis of acute COVID-19 at the point of care: a clinical performance study. Infect Dis Ther. 24, 1–9 (2021). 10.1007/s40121-021-00413-x PMC7904038 33629225

[35] Pray IW, Ford L, Cole D Performance of an antigen-based test for asymptomatic and symptomatic SARS-CoV-2 testing at two university campuses - Wisconsin, September-October 2020. MMWR Morb. Mortal. Wkly Rep. 69(5152), 1642–1647 (2021). 33382679 10.15585/mmwr.mm695152a3 PMC9191905

[36] Dong L, Zhou J, Niu C Highly accurate and sensitive diagnostic detection of SARS-CoV-2 by digital PCR. Talanta. 224, 121726 (2021). 33379001 10.1016/j.talanta.2020.121726 PMC7588801

[37] Beck ET, Paar W, Fojut L, Serwe J, Jahnke RR. Comparison of the Quidel Sofia SARS FIA test to the Hologic Aptima SARS-CoV-2 TMA test for diagnosis of COVID-19 in symptomatic outpatients. J. Clin. Microbiol. 59(2), e02727–02720 (2021). 33239376 10.1128/JCM.02727-20 PMC8111148

[38] Jääskeläinen AE, Ahava MJ, Jokela P Evaluation of three rapid lateral flow antigen detection tests for the diagnosis of SARS-CoV-2 infection. J Clin Virol. 137, 104785 (2021). 33711694 10.1016/j.jcv.2021.104785 PMC7934791

[39] Food and Drug Administration. https://www.fda.gov/medical-devices/coronavirus-disease-2019-covid-19-emergency-use-authorizations-medical-devices/in-vitro-diagnostics-euas-antigen-diagnostic-tests-sars-cov-2

[40] Peeling RW, Holmes KK, Mabey D, Ronald A. Rapid tests for sexually transmitted infections (STIs): the way forward. Sex Transm Infect. 82(Suppl. 5), v1–6 (2006). 17151023 10.1136/sti.2006.024265 PMC2563912

[41] Wu G, Zaman MH. Low-cost tools for diagnosing and monitoring HIV infection in low-resource settings. Bull World Health Organ. 90(12), 914–920 (2012). 23284197 10.2471/BLT.12.102780 PMC3524957

[42] Peeling RW, Olliaro PL, Boeras DI, Fongwen N. Scaling up COVID-19 rapid antigen tests: promises and challenges. Lancet Infect Dis. 23(21), 00048–00047 (2021). 10.1016/S1473-3099(21)00048-7 PMC7906660 33636148

[43] Dinnes J, Deeks JJ, Berhane S Rapid, point-of-care antigen and molecular-based tests for diagnosis of SARS-CoV-2 infection. Cochrane Database Syst Rev. 3, CD013705 (2021). . 33760236 10.1002/14651858.CD013705.pub2 PMC8078597

[44] Hogan CA, Garamani N, Lee AS Comparison of the Accula SARS-CoV-2 Test with a Laboratory-Developed Assay for Detection of SARS-CoV-2 RNA in Clinical Nasopharyngeal Specimens. J. Clin. Microbiol. 58(8), e01072–01020 (2020). 32461285 10.1128/JCM.01072-20 PMC7383558

[45] Eckbo EJ, Locher K, Caza M, Li L, Lavergne V, Charles M. Evaluation of the BioFire^®^ COVID-19 test and Respiratory Panel 2.1 for rapid identification of SARS-CoV-2 in nasopharyngeal swab samples. Diagn. Microbiol. Infect. Dis. 99(3), 115260 (2021). 33340934 10.1016/j.diagmicrobio.2020.115260 PMC7654322

[46] Creager HM, Cabrera B, Schnaubelt A Clinical evaluation of the BioFire^®^ Respiratory Panel 2.1 and detection of SARS-CoV-2. J Clin Virol. 129, 104538 (2020). 32650276 10.1016/j.jcv.2020.104538 PMC7336953

[47] Hansen G, Marino J, Wang ZX Clinical performance of the point-of-care cobas liat for detection of SARS-CoV-2 in 20 minutes: a multicenter study. J. Clin. Microbiol. 59(2), e02811–02820 (2021). 33239382 10.1128/JCM.02811-20 PMC8111162

[48] Donato LJ, Trivedi VA, Stransky AM Evaluation of the Cue Health point-of-care COVID-19 (SARS-CoV-2 nucleic acid amplification) test at a community drive through collection center. Diagn. Microbiol. Infect. Dis. 100(1), 115307 (2021). 33571863 10.1016/j.diagmicrobio.2020.115307 PMC7785428

[49] Cradic K, Lockhart M, Ozbolt P Clinical evaluation and utilization of multiple molecular *in vitro* diagnostic assays for the detection of SARS-CoV-2. Am. J. Clin. Pathol. 154(2), 201–207 (2020). 32462195 10.1093/ajcp/aqaa097 PMC7314271

[50] Fung B, Gopez A, Servellita V Direct comparison of SARS-CoV-2 analytical limits of detection across seven molecular assays. J. Clin. Microbiol. 58(9), e01535–01520 (2020). 32651238 10.1128/JCM.01535-20 PMC7448668

[51] Harrington A, Cox B, Snowdon J Comparison of Abbott ID now and Abbott m2000 methods for the detection of SARS-CoV-2 from nasopharyngeal and nasal swabs from symptomatic patients. J. Clin. Microbiol. 58(8), e00798–00720 (2020). 32327448 10.1128/JCM.00798-20 PMC7383519

[52] Jin R, Pettengill MA, Hartnett NL, Auerbach HE, Peiper SC, Wang Z. Commercial severe acute respiratory syndrome coronavirus 2 (SARS-CoV-2) molecular assays: superior analytical sensitivity of cobas SARS-CoV-2 Relative to NxTAG CoV extended panel and ID NOW COVID-19 Test. Arch. Pathol. Lab. Med. 144(11), 1303–1310 (2020). 32649229 10.5858/arpa.2020-0283-SA

[53] Lephart PR, Bachman MA, LeBar W Comparative study of four SARS-CoV-2 Nucleic Acid Amplification Test (NAAT) platforms demonstrates that ID NOW performance is impaired substantially by patient and specimen type. Diagn. Microbiol. Infect. Dis. 99(1), 115200 (2021). 32980807 10.1016/j.diagmicrobio.2020.115200 PMC7470790

[54] Mitchell SL, George KS. Evaluation of the COVID19 ID NOW EUA assay. J Clin Virol. 128, 104429 (2020). 32425657 10.1016/j.jcv.2020.104429 PMC7227587

[55] Moore NM, Li H, Schejbal D, Lindsley J, Hayden MK. Comparison of two commercial molecular tests and a laboratory-developed modification of the CDC 2019-nCoV reverse transcriptase PCR assay for the detection of SARS-CoV-2. J. Clin. Microbiol. 58(8), e00938–00920 (2020). 32461287 10.1128/JCM.00938-20 PMC7383545

[56] Procop GW, Brock JE, Reineks EZ A comparison of five SARS-CoV-2 molecular assays with clinical correlations. Am. J. Clin. Pathol. 155(1), 69–78 (2021). 33015712 10.1093/ajcp/aqaa181 PMC7665304

[57] Ravi N, Cortade DL, Ng E, Wang SX. Diagnostics for SARS-CoV-2 detection: a comprehensive review of the FDA-EUA COVID-19 testing landscape. Biosens. Bioelectron. 165, 112454 (2020). 32729549 10.1016/j.bios.2020.112454 PMC7368663

[58] Rhoads DD, Cherian SS, Roman K, Stempak LM, Schmotzer CL, Sadri N. Comparison of Abbott ID Now, DiaSorin Simplexa, and CDC FDA emergency use authorization methods for the detection of SARS-CoV-2 from nasopharyngeal and nasal swabs from individuals diagnosed with COVID-19. J. Clin. Microbiol. 58(8), e00760–00720 (2020). 32303564 10.1128/JCM.00760-20 PMC7383529

[59] Serei VD, Cristelli R, Joho K Comparison of abbott ID NOW COVID-19 rapid molecular assay to cepheid xpert xpress SARS-CoV-2 assay in dry nasal swabs. Diagn. Microbiol. Infect. Dis. 99(4), 115208 (2021). 33545455 10.1016/j.diagmicrobio.2020.115208 PMC7493814

[60] Smithgall MC, Scherberkova I, Whittier S, Green DA. Comparison of Cepheid Xpert Xpress and Abbott ID now to Roche cobas for the rapid detection of SARS-CoV-2. J Clin Virol. 128, 104428 (2020). 32434706 10.1016/j.jcv.2020.104428 PMC7217789

[61] Thwe PM, Ren P. How many are we missing with ID NOW COVID-19 assay using direct nasopharyngeal swabs? Findings from a mid-sized academic hospital clinical microbiology laboratory. Diagn. Microbiol. Infect. Dis. 98(2), 115123 (2020). 32673978 10.1016/j.diagmicrobio.2020.115123 PMC7334653

[62] Zhen W, Smith E, Manji R, Schron D, Berry GJ. Clinical evaluation of three sample-to-answer platforms for detection of SARS-CoV-2. J. Clin. Microbiol. 58(8), e00783–00720 (2020). 32332061 10.1128/JCM.00783-20 PMC7383520

[63] Porte L, Legarraga P, Iruretagoyena M Evaluation of two fluorescence immunoassays for the rapid detection of SARS-CoV-2 antigen-new tool to detect infective COVID-19 patients. PeerJ. 9, e10801 (2021). 33552746 10.7717/peerj.10801 PMC7827970

[64] Hou H, Chen J, Wang Y Multicenter evaluation of the Cepheid Xpert Xpress SARS-CoV-2 assay for the detection of SARS-CoV-2 in oropharyngeal swab specimens. J. Clin. Microbiol. 58(8), e01288–01220 (2020). 32503847 10.1128/JCM.01288-20 PMC7383560

[65] Tham JWM, Ng SC, Chai CN Parallel testing of 241 clinical nasopharyngeal swabs for the detection of SARS-CoV-2 virus on the Cepheid Xpert Xpress SARS-CoV-2 and the Roche cobas SARS-CoV-2 assays. Clin Chem Lab Med. 59(2), e45–e48 (2020). 33554503 10.1515/cclm-2020-1338

[66] Wen D, Yang S, Li G, Xuan Q, Guo W, Wu W. Sample-to-answer and routine real-time RRT-PCR: a comparison of different platforms for SARS-CoV-2 detection. J Mol Diagn. 8(21), 00064–00067 (2021). 10.1016/j.jmoldx.2021.02.010 PMC7938732 33706011

[67] Lowe CF, Matic N, Ritchie G Detection of low levels of SARS-CoV-2 RNA from nasopharyngeal swabs using three commercial molecular assays. J Clin Virol. 128, 104387 (2020). 32380382 10.1016/j.jcv.2020.104387 PMC7187880

[68] Kohmer N, Rabenau HF, Hoehl S, Kortenbusch M, Ciesek S, Berger A. Comparative analysis of point-of-care, high-throughput and laboratory-developed SARS-CoV-2 nucleic acid amplification tests (NATs). J. Virol. Methods 291, 114102 (2021). 33607117 10.1016/j.jviromet.2021.114102 PMC7885623

[69] Sieker JT, Horowitz C, Hu CK Analytic sensitivity of 3 nucleic acid detection assays in diagnosis of SARS-CoV-2 infection. J Appl Lab Med. 6(2), 421–428 (2020). 10.1093/jalm/jfaa187 PMC7665530 33674879

[70] Dust K, Hedley A, Nichol K Comparison of commercial assays and laboratory developed tests for detection of SARS-CoV-2. J. Virol. Methods 285, 113970 (2020). 32920028 10.1016/j.jviromet.2020.113970 PMC7482591

[71] Jokela P, Jääskeläinen AE, Jarva H SARS-CoV-2 sample-to-answer nucleic acid testing in a tertiary care emergency department: evaluation and utility. J Clin Virol. 131, 104614 (2020). 32889495 10.1016/j.jcv.2020.104614 PMC7451096

[72] Chen JH, Yip CC, Poon RW Evaluating the use of posterior oropharyngeal saliva in a point-of-care assay for the detection of SARS-CoV-2. Emerg Microbes Infect. 9(1), 1356–1359 (2020). 32459137 10.1080/22221751.2020.1775133 PMC7448919

[73] Falasca F, Sciandra I, Di Carlo D Detection of SARS-COV N2 Gene: very low amounts of viral RNA or false positive? J Clin Virol. 133, 104660 (2020). 33126109 10.1016/j.jcv.2020.104660 PMC7553900

[74] Khoshchehreh M, Wald-Dickler N, Holtom P, Butler-Wu SM. A needle in the haystack? Assessing the significance of envelope (E) gene-negative, nucleocapsid (N2) gene-positive SARS-CoV-2 detection by the Cepheid Xpert Xpress SARS-COV-2 assay. J Clin Virol. 133, 104683 (2020). 33142250 10.1016/j.jcv.2020.104683 PMC7598560

[75] Stevens B, Hogan CA, Sahoo MK Comparison of a point-of-care assay and a high-complexity assay for detection of SARS-CoV-2 RNA. J Appl Lab Med. 5(6), 1307–1312 (2020). 32761092 10.1093/jalm/jfaa135 PMC7454585

[76] Broder K, Babiker A, Myers C Test agreement between Roche cobas 6800 and Cepheid GeneXpert Xpress SARS-CoV-2 assays at high cycle threshold ranges. J. Clin. Microbiol. 58(8), e01187–01120 (2020). 32444354 10.1128/JCM.01187-20 PMC7383541

[77] Goldenberger D, Leuzinger K, Sogaard KK Brief validation of the novel GeneXpert Xpress SARS-CoV-2 PCR assay. J. Virol. Methods 284, 113925 (2020). 32659240 10.1016/j.jviromet.2020.113925 PMC7351036

[78] Lieberman JA, Pepper G, Naccache SN, Huang ML, Jerome KR, Greninger AL. Comparison of commercially available and laboratory-developed assays for *in vitro* detection of SARS-CoV-2 in clinical laboratories. J. Clin. Microbiol. 58(8), e00821–00820 (2020). 32350048 10.1128/JCM.00821-20 PMC7383518

[79] Loeffelholz MJ, Alland D, Butler-Wu SM Multicenter evaluation of the Cepheid Xpert Xpress SARS-CoV-2 test. J. Clin. Microbiol. 58(8), e00926–00920 (2020). 32366669 10.1128/JCM.00926-20 PMC7383535

[80] Moran A, Beavis KG, Matushek SM Detection of SARS-CoV-2 by use of the Cepheid Xpert Xpress SARS-CoV-2 and Roche cobas SARS-CoV-2 assays. J. Clin. Microbiol. 58(8), e00772–00720 (2020). 32303565 10.1128/JCM.00772-20 PMC7383516

[81] Wolters F, van de Bovenkamp J, van den Bosch B Multi-center evaluation of Cepheid Xpert^®^ Xpress SARS-CoV-2 point-of-care test during the SARS-CoV-2 pandemic. J Clin Virol. 128, 104426 (2020). 32417674 10.1016/j.jcv.2020.104426 PMC7211760

[82] Navarathna DH, Sharp S, Lukey J Understanding false positives and the detection of SARS-CoV-2 using the Cepheid Xpert Xpress SARS-CoV-2 and BD MAX SARS-CoV-2 assays. Diagn. Microbiol. Infect. Dis. 100(1), 115334 (2021). 33571862 10.1016/j.diagmicrobio.2021.115334 PMC7987987

[83] Yoon SH, Yang S, Cho H, Eun S, Koo CM, Kim MK. Point-of-care testing for the detection of SARS-CoV-2: a systematic review and meta-analysis. Eur Rev Med Pharmacol Sci. 25(1), 503–517 (2021). 33506942 10.26355/eurrev_202101_24422

[84] Tworek JA, Khan F, Sekedat MD, Scheidel C, Malani AN. The utility of rapid nucleic acid amplification testing to triage symptomatic patients and to screen asymptomatic preprocedure patients for SARS-CoV-2. Open Forum Infect Dis. 8(1), ofaa607 (2021). 33506068 10.1093/ofid/ofaa607 PMC7798501

[85] Leung EC, Chow VC, Lee MK, Tang KP, Li DK, Lai RW. Evaluation of the Xpert Xpress SARS-CoV-2/Flu/RSV assay for simultaneous detection of SARS-CoV-2, influenza A and B viruses, and respiratory syncytial virus in nasopharyngeal specimens. J. Clin. Microbiol. 59(4), e02965–02920 (2021). 33436456 10.1128/JCM.02965-20 PMC8092745

[86] Mostafa HH, Carroll KC, Hicken R Multicenter evaluation of the Cepheid Xpert Xpress SARS-CoV-2/Flu/RSV test. J. Clin. Microbiol. 59(3), e02955–02920 (2021). 33298613 10.1128/JCM.02955-20 PMC8106732

[87] Food and Drug Administration. https://www.fda.gov/medical-devices/coronavirus-disease-2019-covid-19-emergency-use-authorizations-medical-devices/in-vitro-diagnostics-euas-molecular-diagnostic-tests-sars-cov-2#individual-molecular

[88] Basu A, Zinger T, Inglima K Performance of Abbott ID Now COVID-19 rapid nucleic acid amplification test using nasopharyngeal swabs transported in viral transport media and dry nasal swabs in a New York City Academic Institution. J. Clin. Microbiol. 58(8), e01136–01120 (2020). 32471894 10.1128/JCM.01136-20 PMC7383552

[89] Hinson JS, Rothman RE, Carroll K Targeted rapid testing for SARS-CoV-2 in the emergency department is associated with large reductions in uninfected patient exposure time. J. Hosp. Infect. 107, 35–39 (2021). 33038435 10.1016/j.jhin.2020.09.035 PMC7538869

[90] Brendish NJ, Poole S, Naidu VV Clinical impact of molecular point-of-care testing for suspected COVID-19 in hospital (COV-19POC): a prospective, interventional, non-randomised, controlled study. The Lancet Respiratory Medicine 8(12), 1192–1200 (2020). 33038974 10.1016/S2213-2600(20)30454-9 PMC7544498

[91] Collier DA, Assennato SM, Warne B Point of care nucleic acid testing for SARS-CoV-2 in hospitalized patients: a clinical validation trial and implementation study. Cell Rep Med. 1(5), 100062 (2020). 32838340 10.1016/j.xcrm.2020.100062 PMC7362826

[92] Hengel B, Causer L, Matthews S A decentralised point-of-care testing model to address inequities in the COVID-19 response. Lancet Infect Dis. 23(20), 30859–30858 (2020). 10.1016/S1473-3099(20)30859-8 PMC7758179 33357517

[93] Procop GW, Kadkhoda K, Rhoads DD, Gordon SG, Reddy AJ. Home testing for COVID-19: benefits and limitations. Cleve. Clin. J. Med. 1(10), (2021). 10.3949/ccjm.88a.ccc071 33579779

[94] Ibitoye M, Frasca T, Giguere R, Carballo-Diéguez A. Home testing past, present and future: lessons learned and implications for HIV home tests. AIDS Behav. 18(5), 933–949 (2014). . 24281697 10.1007/s10461-013-0668-9 PMC3988264

[95] Minichiello A, Swab M, Chongo M HIV point-of-care testing in Canadian settings: a scoping review. Front Public Health. 5, 76 (2017). 28459048 10.3389/fpubh.2017.00076 PMC5394765

[96] Faust L, Zimmer AJ, Kohli M SARS-CoV-2 testing in low- and middle-income countries: availability and affordability in the private health sector. Microbes Infect. 22(10), 511–514 (2020). 33065265 10.1016/j.micinf.2020.10.005 PMC7553871

[97] Benirschke RC, McElvania E, Thomson RB Jr, Kaul KL, Das S. Clinical impact of rapid point-of-care PCR influenza testing in an urgent care setting: a single-center study. J. Clin. Microbiol. 57(3), e01281–01218 (2019). 10.1128/JCM.01281-18 PMC6425177 30602445

[98] Ahmadivand A, Gerislioglu B, Ramezani Z, Kaushik A, Manickam P, Ghoreishi SA. Functionalized terahertz plasmonic metasensors: femtomolar-level detection of SARS-CoV-2 spike proteins. Biosens. Bioelectron. 177, 112971 (2021). 33434777 10.1016/j.bios.2021.112971 PMC7787065

[99] Jain S, Nehra M, Kumar R Internet of medical things (IoMT)-integrated biosensors for point-of-care testing of infectious diseases. Biosens. Bioelectron. 179, 113074 (2021). 33596516 10.1016/j.bios.2021.113074 PMC7866895

[100] Kaushik A. Manipulative magnetic nanomedicine: the future of COVID-19 pandemic/endemic therapy. Expert Opin Drug Deliv. 14, 1–4 (2020). 10.1080/17425247.2021.1860938 33307877

[101] Cao L, Cui X, Hu J Advances in digital polymerase chain reaction (dPCR) and its emerging biomedical applications. Biosens. Bioelectron. 90, 459–474 (2017). 27818047 10.1016/j.bios.2016.09.082

